# Gas Flow Shaping via Novel Modular Nozzle System (MoNoS) Augments kINPen-Mediated Toxicity and Immunogenicity in Tumor Organoids

**DOI:** 10.3390/cancers15041254

**Published:** 2023-02-16

**Authors:** Julia Berner, Lea Miebach, Luise Herold, Hans Höft, Torsten Gerling, Philipp Mattern, Sander Bekeschus

**Affiliations:** 1 ZIK *plasmatis*, Leibniz Institute for Plasma Science and Technology (INP), Felix-Hausdorff-Straße 2, 17489 Greifswald, Germany; 2 Department of Oral, Maxillofacial and Plastic Surgery, Greifswald University Medical Center, Ferdinand-Sauerbruch-Straße, 17475 Greifswald, Germany; 3 Department of General, Visceral, Thoracic and Vascular Surgery, Greifswald University Medical Center, Ferdinand-Sauerbruch-Straße, 17475 Greifswald, Germany; 4 Department of Plasma Diagnostics, Leibniz Institute for Plasma Science and Technology (INP), Felix-Hausdorff-Straße 2, 17489 Greifswald, Germany; 5 Diabetes Competence Centre Karlsburg (KDK), Leibniz Institute for Plasma Science and Technology (INP), Greifswalder Straße 11, 17495 Karlsburg, Germany

**Keywords:** APPJ, CAP, HET-CAM, in ovo, MoNoS, reactive oxygen species, TUM-CAM

## Abstract

**Simple Summary:**

Cancer is a devastating disease. New treatment avenues are demanded to promote successful and safe cancer therapies. Gas plasma is a novel tool recently promoted for cancer treatment. This so-called fourth state of matter is known in its hotter forms, such as fire and lightning. Technology leap innovations enabled the usage of gas plasma for medical purposes. In laboratory models, gas plasma has shown promising antitumor effects in several types of cancer. One particularly successful gas plasma device type is called jet plasma. We here attempted to optimize those by testing two adapters mountable on plasma jet devices, which have two functions. One is to increase the amount of ambient air, similar to a turbo coming close to the plasma jet, to produce more free radicals within the same time for anticancer treatment. The second is to add a filter with varying porosity between the plasma jet and the treatment target. This increases the area of free radical deposition, potentially enabling larger skin or tumor treatment areas compared to the focused treatment area of the plasma jet alone. We here provide evidence that such a filter enhanced the antitumor effects of a certified argon plasma jet.

**Abstract:**

Medical gas plasma is an experimental technology for anticancer therapy. Here, partial gas ionization yielded reactive oxygen and nitrogen species, placing the technique at the heart of applied redox biomedicine. Especially with the gas plasma jet kINPen, anti-tumor efficacy was demonstrated. This study aimed to examine the potential of using passive flow shaping to enhance the medical benefits of atmospheric plasma jets (APPJ). We used an in-house developed, proprietary Modular Nozzle System (MoNoS; patent-pending) to modify the flow properties of a kINPen. MoNoS increased the nominal plasma jet-derived reactive species deposition area and stabilized the air-plasma ratio within the active plasma zone while shielding it from external flow disturbances or gas impurities. At modest flow rates, dynamic pressure reduction (DPR) adapters did not augment reactive species deposition in liquids or tumor cell killing. However, MoNoS operated at kINPen standard argon fluxes significantly improved cancer organoid growth reduction and increased tumor immunogenicity, as seen by elevated calreticulin and heat-shock protein expression, along with a significantly spurred cytokine secretion profile. Moreover, the safe application of MoNoS gas plasma jet adapters was confirmed by their similar-to-superior safety profiles assessed in the hen’s egg chorioallantoic membrane (HET-CAM) coagulation and scar formation irritation assay.

## 1. Introduction

Reactive oxygen and nitrogen species (abbreviated as ROS because RNS also contain oxygen) are key players in physiological signaling pathways. The uncontrolled activation pattern of the latter is among the hallmarks of cancer [1]. For instance, hydrogen peroxide (H_2_O_2_) can deregulate phosphoinositide 3-kinase/Akt signaling, followed by increased cell proliferation and apoptosis inhibition [2]. Additionally, reactive oxygen species (ROS)-dependent regulation of calcium channels enhances cellular proliferation [3]. Due to their aggressive growth, tumor cells exhibit a deregulated metabolism with shifts in carbon consumption, generating even higher levels of growth-stimulating ROS via pyruvate oxidation in mitochondria [4]. In this light, early research in redox oncology focused on ROS as major contributors to malignant transformation, fostering the preclinical investigation of antioxidant therapies for oncological treatment regimes. Strikingly, antioxidant treatment for limiting tumor development did not meet expectations. Several studies even suggested ROS limitation promoted cancer development and revealed the role of ROS-dependent cell death signaling upon increased oxidative damage [5,6]. Simultaneously, many chemotherapeutic drugs and radiotherapy were found to induce oxidative distress and apoptosis [7,8].

Promising findings on antitumoral effects of reactive species stimulated the successful development of ROS-based onco-therapies, such as photodynamic therapy (PDT) [9] and medical gas plasma technology [10]. While the former is mainly based on the local formation of singlet delta oxygen, gas plasmas are exceptional in simultaneously generating a plethora of highly reactive ROS [11]. The potential of medical gas plasmas to contribute to the oncologists’ toolbox has been outlined by extensive preclinical research in recent years. A clinical case study conducted at the Greifswald University Medical Center (Germany) showed that head and neck cancer patients benefited from palliative treatment using the kINPen Med [12]. However, one of the jet’s shortcomings is the small treatment area covered. This leads to extensive gas plasma treatment times for covering large tissue areas. This potentially limits the patients’ therapy compliance and is associated with higher costs. In addition, gas plasma jets, especially when operated with relatively heavy argon compared to light helium, have a strong momentum flux (physical pressure on the target), making their investigation in regular preclinical studies in vitro challenging due to potential liquid displacement in, e.g., tumor cell cultures. In addition, at high feed gas fluxes, direct contact between the gas plasma jet and a liquid target in vitro is not possible [13].

A Modular Nozzle System (MoNoS; patent pending) was developed in-house to overcome these limitations. These 3D-printed ceramic systems can be adapted to the required specifications, but their potential in biomedical research, including oncology, has not yet been addressed. To this end, we performed a head-to-head comparison of a standard kINPen gas plasma jet treatment to a MoNoS-modified kINPen gas plasma jet treatment. Two MoNoS adapters were used, one with a low and one with a high dynamic pressure reduction (DPR). For comparison, we characterized the gas plasma-derived ROS chemistry in liquids and the anti-tumor effects in vitro and in solid, vascularized tumors grown on the chorioallantoic membrane (CAM) of fertilized chicken embryos in ovo [14], also focusing on the immunogenicity of the treatment. In addition, the treatment’s safety was evaluated using the modified hen’s egg chorioallantoic membrane (HET-CAM) irritation assay [15]. Our results indicate a highly promising role of MoNoS adapters in enhancing the effects of the approved gas plasma jet kINPen without interfering with its electrical operation.

## 2. Experimental Section

### 2.1. Cell Culture

The murine luciferase-expressing carcinoma cells CT26-luc (UBIGENE; catalog number YC-B003-Luc-P) and human pancreatic cancer cell lines PANC-1 (ACC: 783) were cultured in Roswell Park Memorial Institute (RPMI) 1640 medium supplemented with 10% fetal bovine serum, 1% glutamine, and 1% penicillin/streptomycin (all Sigma-Aldrich, Taufkirchen, Germany) at 37 °C and 5% CO_2_ in 95% humidified air (*v*/*v*). Twenty-four hours before gas plasma treatment, cells were seeded at a density of 0.5 × 10^5^ cells per well (24-well flat bottom plates; Sarstedt) in 500 µL of fully supplemented cell culture medium.

### 2.2. The Modular Nozzle System (MoNoS)

**Figure 1:**
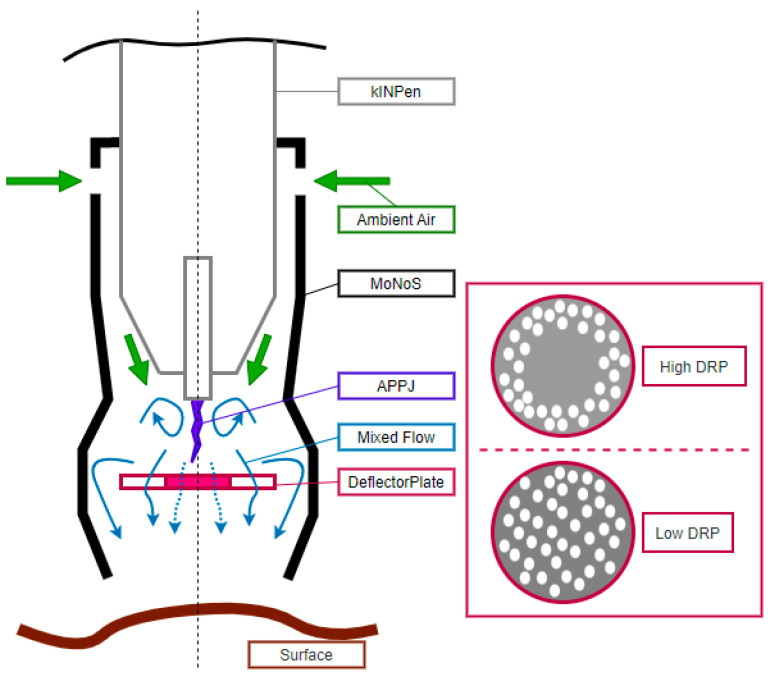
**Half-section of an exemplary MoNoS adapter (black) surrounding the kINPen (gray).** The argon APPJ (effluent indicated in purple) drives a pressure gradient that sucks in ambient air (green arrow, indicating flow direction) at a constant rate, stabilizing the air-plasma ratio at the mixing zone (blue swirl arrow) around the effluent, and, thus, ROS production. A porous deflector plate is used for dynamic pressure reduction (DPR) of the jet. The amount of pressure reduction is controlled by the porosity of the plate. Two versions were used: a partially perforated (high DPR) disk with a fully solid center region (no dotted arrow center outflow) and a fully perforated (low DPR) disk (outflow includes dotted arrows). This results in a homogenized, settled, and mixed outflow (blue arrows) with a strongly reduced axial flow momentum onto the treated surface (brown).

The Modular Nozzle System (MoNoS) was developed in-house to overcome the limitations and shortcomings of plasma jets in a case-adaptive way (Figure 1). Each adapter is self-priming. Ambient air is sucked into the adapter at a constant flow rate, driven by a pressure gradient induced by the APPJ. This stabilizes the air-to-feed gas (argon) ratio within the active plasma zone while shielding it from external flow disturbances or gas impurities, all being crucial for ROS production. Furthermore, the dynamic pressure of the jet (the pressure onto the treated surface) is decreased using a deflector plate downstream of the jet. The porosity of the plate controls the extent of dynamic pressure reduction (DPR). Another feature is its ability to diffuse the plasma-gas composition at the outlet (the pinpoint nature of a non-conductive gas plasma jet can be fanned out to extend the treated surface area). The system is designed to be 3D printable, enabling different material options, from single-use plastics to autoclavable ceramics. Two adapters were used in this study. Both are identical in geometry, thus self-priming with comparable auxiliary gas flow. Only the porosity of the deflector plate was modified to achieve two different dynamic pressure reduction levels. The ^low^DPR version uses a fully perforated disk, while the ^high^DPR version uses a partially perforated disk with a solid center region. This leads to stronger dynamic pressure reduction and mixing effects for the ^high^DPR version compared to the ^low^DPR version. For this study, the MoNoS adapters were made entirely out of ceramic (Al_2_O_3_) to offer the best chemically inert properties, thus being reusable by disinfecting the system after clinical application. We here demonstrated beneficial anticancer effects of passive APPJ flow shaping, and studying the physical processes of the latter will be a subject of future investigations.

### 2.3. Gas Plasma Jet kINPen and Treatment

This study used a research version of the atmospheric pressure gas plasma jet kINPen (neoplas med, Greifswald, Germany) for gas plasma treatment. It is similar to the kINPen MED approved as a medical class IIa device in Europe [15]. Its technical and chemical properties have been extensively described before [16]. The jet was operated at different flow rates between 1 and 5 standard liters per minute (slm) with argon gas (99.999% purity; Air Liquide, Bremen, Germany) excited at the electrode within the kINPen at 1 MHz and a dissipated power of 1 W. 3D-printed ceramic diffusor adapters (MoNoS) were installed on the kINPen head. Therapeutic efficacy was compared to regular kINPen treatment (ArP; i.e., without an adapter). In vitro treatments were performed with cell culture medium covering the cells. Using a computer-controlled and motorized xyz stage (CNC, Espelkamp, Germany), the kINPen was hovered over the center of each well at 12 mm distance between the nozzle and liquid surface or at 18 mm in the case of the adapters. For gas plasma treatment in ovo, the jet was positioned in direct contact with the target surface (conducting mode) [13] or at the closest possible distance in the case of the adapters, which was less than 5 mm.

### 2.4. Profiling of Reactive Oxygen and Nitrogen Deposition

For characterization of ROS deposition, 1 mL PBS was exposed to gas plasma in 24-well plates (Sarstedt, Germany) and analyzed immediately after treatment. Evaporated volume was replaced with equal amounts of double distilled water to maintain iso-osmolarity. The evaporated volume was determined by weight. Changes in pH were measured using a pH meter (Mettler-Toledo, Berlin, Germany). Relative detection of peroxynitrite (ONOO^−^) and hydroxyl radical (^.^OH), including or without hypochlorous acid (HOCl), was performed using the fluorescent redox probes aminophenyl fluoresceine (APF) and hydroxyphenyl fluoresceine (HPF; both Enzo Life Sciences, Lörrach, Germany), respectively. Absolute quantification of hydrogen peroxide (H_2_O_2_) formation was conducted using the Amplex Ultra Red assay (Thermo Fisher Scientific, Dreieich, Germany). Nitric oxide (NO) was detected using the fluorescent redox probe 4-amino-5-methylamino-2′,7′-dichlorofluorescein (DAF; Thermo Fisher Scientific). Assessment of nitrite (NO_2_^−^) and nitrate (NO_3_^−^) generation was performed using the Griess assay (Biomol, Hamburg, Germany). Absolute concentrations were calculated against a standard curve.

### 2.5. Metabolic Activity

Metabolic activity was determined by adding 100 µM 7-hydroxy-3H-enoxacin-3-on-10-oxid (resazurin; Alfa Aesar, Kandel, Germany) to the cells 20 h after treatment. Viable cells metabolize non-fluorescent resazurin to fluorescent resorufin in a NADPH/H+-dependent reaction. After 4 h of incubation, fluorescence was measured at λex = 535 nm and λem = 590 nm using a multimode plate reader (F200; Tecan, Männdorf, Switzerland).

### 2.6. Bioluminescence

Bioluminescence was used to assess cellular viability *in vitro*. Briefly, 25 ng/mL luciferin (PerkinElmer, Hamburg, Germany) was added to the cells. Luminescence was measured using a multimode plate reader (M200; Tecan). For luminescence measurements ex ovo, 100 µL of 2.5 µg/mL luciferin was added on top of the tumor. Luminescence was measured immediately over a time period of 10 min.

### 2.7. Flow Cytometry

Cellular viability after gas plasma treatment in vitro was assessed using flow cytometry. For this, cells were stained at 37 °C for 30 min with 1 µM 4′, 6-diamide-2-phenylindole (DAPI; BioLegend, Amsterdam, The Netherlands) to identify terminally dead cells and 0.5 µM active caspases 3/7 detection reagent (Thermo Fisher Scientific) for labeling apoptotic cells. After washing, cells were acquired using flow cytometry (CytoFLEX S; Beckman-Coulter, Krefeld, Germany). Gating and quantification of mean fluorescence intensities were performed using Kaluza 2.2 analysis software (Beckman-Coulter).

### 2.8. In Ovo Model

The in ovo model was used to evaluate the anti-tumor activity and irritation potential of the MoNoS-complemented gas plasma exposure. To assess the cytotoxic and immunogenic impact of the different treatment modes, the tumor-chorioallantoic membrane (TUM-CAM) assay was performed as previously described [14]. Specific-pathogen-free (SPF) eggs (Valo BioMEdia, Osterholz-Scharmbeck, Germany) were bred for 6 days (60% humidity, 37 °C) before the eggs’ pointed poles were punctured at embryonic day (ED) 7. After 24 h of incubation, eggs were opened, and 1 × 10^6^ tumor cells in 15 µL Matrigel were seeded in a plastic ring on the CAM. Gas plasma treatment was performed at ED 10. Eggs were incubated for another 4 days before solid tumors were excised and weighed. Tumors were digested using a specific tumor dissociation kit and the gentleMACS Octo dissociator (Miltenyi-Biotec, Teterow, Germany) to prepare viable single-cell suspensions. Supernatants of digested tumors were collected for secretion profile analysis. Cells were stained with anti-mouse monoclonal antibodies targeting calreticulin (CRT; Alexa Fluor 594; BioTechne, Wiesbaden, Germany), heat-shock protein (HSP) 70 (allophycocyanin; BioTechne), HSP90 (phycoerythrin; EnzoLifeScience, Farmingdale, NY, USA), major histocompatibility complex (MHC) I (Brilliant Ultra Violet 661; Becton-Dickinson Biosciences, Heidelberg, Germany) and anti-chicken MHC I (fluorescein isothiocyanate; Biozol, Hamburg, Germany) for flow cytometric analysis (CytoFLEX LX; Beckman-Coulter). The hen’s egg-chorioallantoic membrane test (HET-CAM) was utilized to determine the irritation potential and safety of the different MoNoS adapters. For this, the CAM was exposed to gas plasma at ED 8 directly after opening the eggshell. Macroscopic photographic images of the CAM were taken before and 24 h and 48 h post-treatment. An in-house scoring system was used to classify the response patterns in a blinded evaluation.

### 2.9. Chemokine and Cytokine Release Profiling

Chemokine and cytokine analysis of in ovo tumor supernatants was performed using a bead-based sandwich multi-analyte assay (LegendPLEX; BioLegend) as recently described [17]. The assay panel contained beads targeted against epithelial-derived neutrophil-activating protein (ENA) 78, granulocyte colony-stimulating factor (G-CSF), granulocyte-macrophage colony-stimulating factor (GM CSF), growth-regulated oncogene (GRO) α, hepatocyte growth factor (HGF), interferon (IFN) γ, interleukin (IL)1β, IL2, IL6, IL18, interferon-gamma induced protein (IP) 10, platelet-derived growth factor-AA (PDGF-AA), and vascular endothelial growth factor (VEGF). Beads were labeled with fluorescent detection antibodies, and samples were acquired using flow cytometry (CytoFLEX LX). Absolute concentrations of respective analytes were calculated against a standard curve using specific data analysis software (BioLegend).

### 2.10. Software and Statistical Analysis

Data normalization was performed using Excel 2021 (Microsoft, Redmond, WA, USA). Data analysis and graphing were performed utilizing Prism 9.5.0 (GraphPad Software, San Diego, CA, USA). One-way analysis of variances or t-test was performed to determine the degree of statistical significance between groups. The level of significance was indicated as follows: *p* < 0.05 (*), *p* < 0.01 (**), and *p* < 0.001 (***).

## 3. Results

### 3.1. MoNoS-Complemented kINPen Treatment at Modest Feed Gas Fluxes Yielded Reduced ROS Deposition in Liquids

**Figure 2:**
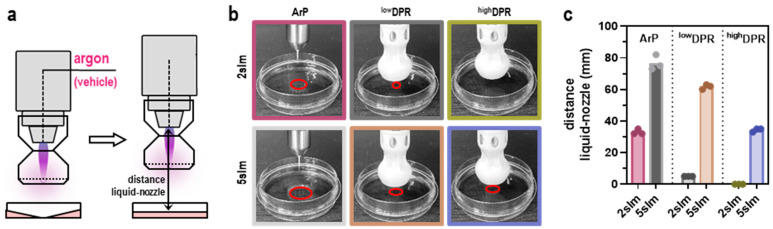
**Medical gas plasma jet kINPen and MoNoS adapters.** (**a**) schematic overview of gas plasma treatment using the MoNoS adapters; (**b**) representative images of gas plasma treatment without (ArP) and with DPR (red circles indicate the region of volume displacement); (**c**) closest possible distance of liquid to the nozzle (without liquid spillage) measured at different gas flow rates and with different adapters. Bar graphs show mean and individual values. slm = standard liters per minute; ArP = argon plasma (typical setup without adapter); ^low^DPR = low dynamic pressure reduction; ^high^DPR = high dynamic pressure reduction.

MoNoS adapters increase the nominal gas plasma jet-derived ROS deposition area by stabilizing the air–plasma ratio within the active plasma zone and guiding the mixed flow through a well-defined outlet cross-section. In parallel, MoNoS gas plasma jet operation reduces the jet’s impact (dynamic pressure) on the treated surface. The extent of gas flow pressure breakdown was indirectly measured by assessing liquid displacement of regular kINPen treatment (ArP) compared to the MoNoS system. Using a computer-controlled xyz-stage, the jet was moved in the vertical direction (Figure 2a), and the height was measured until liquid displacement could no longer be observed (Figure 2b). Since the MoNoS system reduces the plasma gas flow (dynamic pressure, thus the direct pressure) on the liquid surface, utilizing MoNoS adapters significantly decreased the jet-to-target distance of the kINPen for achieving minimal liquid perturbations (Figure 2c).

**Figure 3:**
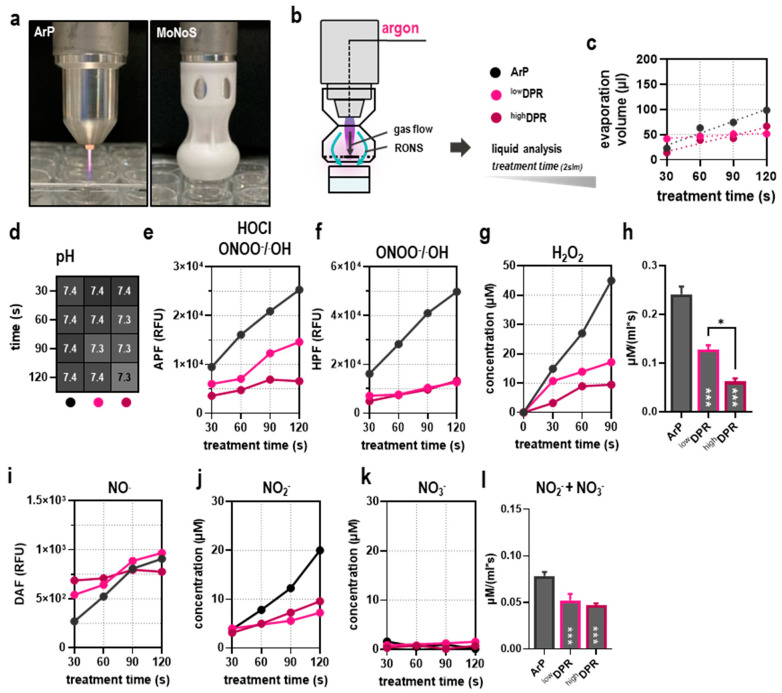
**Reactive species profiling of MoNoS-complemented gas plasma treatment.** (**a**) representative images of MoNoS-complemented gas plasma treatment and the surface profiles of the different adapters utilized; (**b**) schematic overview of liquid analysis assessed at varying treatment times; (**c**) liquid evaporation in dependence on treatment time; (**d**) changes in pH in dependence on treatment time; (**e**,**f**) detection of short-lived ROS using the fluorescent redox probes aminophenyl fluoresceine (APF, (**e**)) and hydroxyphenyl fluoresceine (HPF, (**f**)) at varying treatment times; (**g**,**h**) quantification of hydrogen peroxide (H_2_O_2_) deposition in dependence of treatment time (**g**) and relative H_2_O_2_ deposition per milliliter and second with the different adapters (**h**); (**i**) detection of nitric monoxide (NO) at varying treatment times; (**j**,**k**) quantification of nitrite (NO_2_^−^, (**j**)) and nitrate (NO_3_^−^, (**k**)) deposition with varying treatment times; (**l**) relative total NO_2_^−^ and NO_3_^−^ deposition with the different adapters. XY plots show mean. Bar graphs show mean + standard error of the mean (SEM). Statistical analysis was performed using one-way analysis of variance (ANOVA) (* *p* < 0.05, *** *p* < 0.001). ns = non-significant; ROS = reactive oxygen and nitrogen species; slm = standard liters per minute; RFU = relative fluorescence units; ArP = argon plasma (typical setup without adapter).

Therefore, the adapters would allow kINPen treatment of pressure-sensitive surfaces. The effect was larger for MoNoS II over MoNoS I due to the non-permeable central region of the deflector plate. Next, ROS deposited in liquids were compared between the respective kINPen treatment regimens (Figure 3a). Liquid analysis was performed immediately after gas plasma exposure (Figure 3b). As expected, liquid evaporation increased with prolonged treatment times. In comparison, MoNoS-complemented gas plasma exposure showed approximately halved evaporation rates compared to typical kINPen treatment (ArP, i.e., without adapter) (Figure 3c). Major changes in pH could not be observed in any condition tested (Figure 3d). The kINPen gas plasma operation generated a multitude of ROS. Relative assessment of peroxynitrite (ONOO^−^) and/or hydroxyl radicals (^.^OH) including (Figure 3e) or without (Figure 3f) hypochlorous acid (HOCl) revealed a linear increase with prolonged kINPen treatment times, and ROS levels were significantly reduced when MoNoS adapters were installed. It must be mentioned that only modest feed gas fluxes (2 slm) were used for these treatments. This facilitated comparing MoNoS-complemented plasma treatment with typical kINPen plasma treatment in vitro. At gas fluxes higher than 2 slm, liquid target exposure to the kINPen was impaired due to liquid displacement (Figure 2c). Similarly to short-lived species levels, H_2_O_2_ formation also was diminished when using MoNoS adapters compared with regular kINPen treatment without adapters (Figure 3g). ROS deposition rates seem to show a dependency on momentum, i.e., reduced momentum led to lower H_2_O_2_ rates (Figure 3h). Strikingly, with MoNoS adapters, nitric oxide (NO) generation was enhanced compared to typical kINPen treatments at short exposure times (Figure 3i), although no increased formation of nitrite (NO_2_^−^; Figure 3j) or nitrate (NO_3_^−^; Figure 3k) was found in the MoNoS-complemented conditions (Figure 3l). Altogether, reduced ROS and RNS production was found in liquids upon MoNoS-complemented gas plasma treatment if operated at modest (2 slm) feed gas fluxes.

### 3.2. MoNoS-Complemented kINPen Treatment at Modest Gas Fluxes Does Not Augment Tumor Toxicity In Vitro and In Ovo

**Figure 4:**
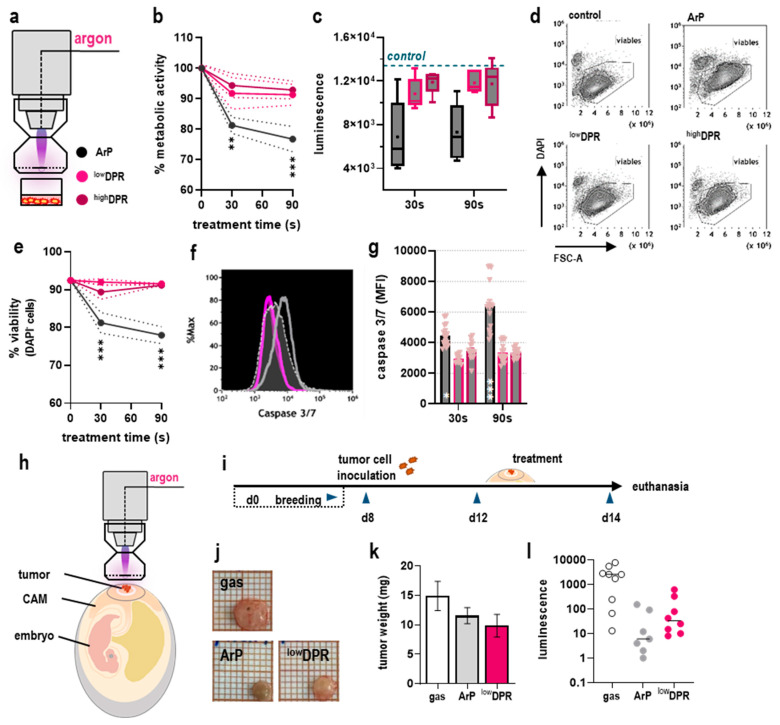
**Anti-tumor activity of MoNoS-complemented gas plasma treatment is not superior to typical kINPen treatment at modest flow rates.** (**a**) schematic overview of gas plasma treatment with cells in cell culture media; (**b**) metabolic activity at 24 h as a function of exposure time; (**c**) tumor cell luminescence after gas plasma treatment for 30 s and 90 s with ArP, ^low^DPR, and ^high^DPR; (**d**,**e**) representative flow cytometric dot plots of DAPI and forward scatter (**d**) and quantification of viable (DAPI^-^) cells (**e**); (**f**,**g**) representative flow cytometric histogram of caspase 3/7 activation (**f**) and quantification of activated caspase 3/7 fluorescence (**g**); (**h**,**i**) schematic overview of gas plasma treatment in ovo (**h**) and the experimental procedure (**i**); (**j**) representative tumor images; (**k**) tumor weights and (**l**) tumor luminescence (gas, *n* = 9; ArP, *n* = 7; ^low^DPR, *n* = 8). Statistical analysis was performed using one-way analysis of variance (ANOVA) (* *p* < 0.05, ** *p* < 0.01, *** *p* < 0.001). CAM = chorioallantoic-membrane; MFI = mean fluorescence intensity; ArP = argon plasma (typical setup without adapter).

**Figure A1:**
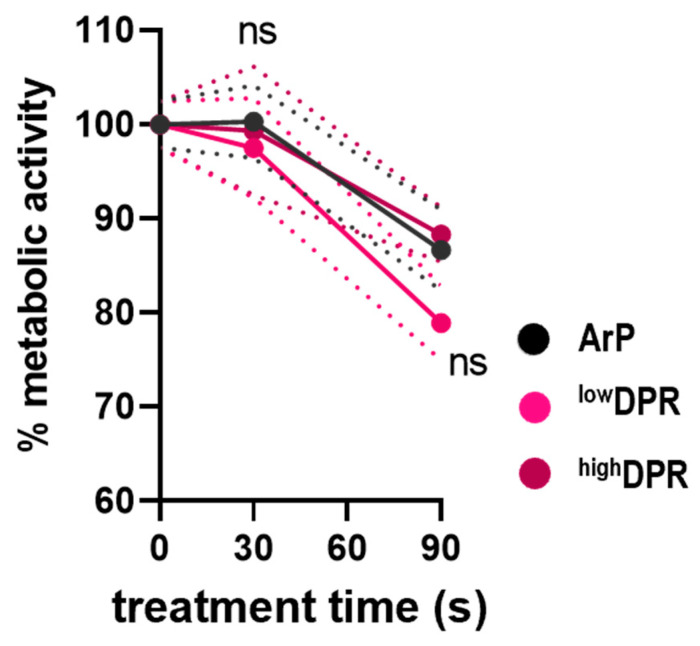
**Metabolic activity after gas plasma treatment.** Metabolic activity of CT26-luc murine colon carcinoma cells 24 h after exposure to argon plasma (ArP) and additional flow-shaping adapters. Statistical analysis was performed using one-way analysis of variance (ANOVA). ns = non-significant. ^low^DPR = low dynamic pressure reduction; ^high^DPR = high dynamic pressure reduction.

Gas plasmas can elicit oxidative distress in tumor cells, leading to irreversible damage of cellular biomolecules and induction of cell death. We examined the performance of MoNoS adapters (Figure 4a) at modest feed gas fluxes (2 slm). As mentioned above, this was done to compare MoNoS with regular kINPen treatment, which can hardly be performed at 5 slm *in vitro*. Regular kINPen treatment (ArP) significantly reduced the metabolic activity of cells in a treatment time-dependent manner compared to untreated controls, while MoNoS-complemented gas plasma treatment did so to a lesser extent (Figure 4b). This relation was also observed in luminescence measurements (Figure 4c) and analysis of terminal cell death (Figure 4d) quantitatively assessed using flow cytometry (Figure 4e). Cell death was characterized by increased activation of effector caspases 3 and 7 (Figure 4f) involved in the terminal pathways of apoptotic cascades (Figure 4g). However, in less sensitive Panc-1 cancer cells, toxicity differences between gas plasma exposure with or without ^high^DPR were not apparent (Figure A1). In general, the translational relevance of in vitro results in gas plasma oncological research is limited by the given reaction kinetics of short-lived reactive species in bulk liquids surrounding the cells. Mammalian in vivo models remain state-of-the-art but are often limited by time, costs, and ethical constraints. Therefore, the in ovo model has been successfully used to grow 3D tumor organoids on the well-vascularized chorioallantoic membrane of chicken embryos and was chosen as a semi-in vivo model for treatment evaluation in our study (Figure 4h). Following an initial breeding period, tumor cells were inoculated on the CAM at embryonic day (ED) 8, forming solid, matrix-producing tumors until ED 12, when kINPen gas plasma treatment (2slm) was performed with or without MoNoS adapters. The eggs were sacrificed on ED 14 (Figure 4i), followed by careful tumor excision (Figure 4j). A superior tumor reduction by MoNoS-complemented kINPen treatment could not be observed over the argon gas-treated controls (Figure 4k), which was confirmed by luminescence measurements (Figure 4l).

### 3.3. MoNoS-Complemented kINPen Treatment at 5 slm Augments Tumor Toxicity

**Figure 5:**
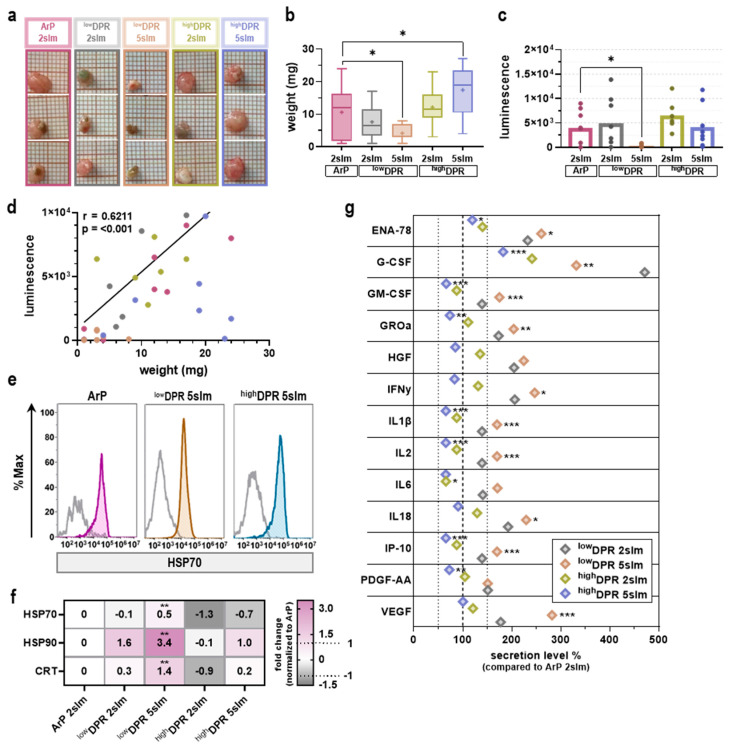
**Anti-tumor activity of MoNoS-complemented gas plasma treatment outperforms typical kINPen treatment at high gas flow rates.** (**a**) representative images of in ovo grown tumors; (**b**) tumor weights and; (**c**) luminescence (ArP, *n* = 6; ^low^DPR 2slm, *n* = 7; ^low^DPR 5slm, *n* = 4; ^high^DPR 2slm, *n* = 6; ^high^DPR 5slm, *n* = 7); (**d**) Pearson’s correlation of tumor weight and luminescence; (**e**) representative flow cytometric histograms showing HSP70 expression; (**f**) heat map showing relative expression of HSP70, HSP90, and CRT after MoNoS-complemented gas plasma exposure normalized to ArP; (**g**) chemokine and cytokine release of in ovo grown tumors normalized to ArP. Data are given as box plots or bar graphs showing mean and individual values. Statistical analysis was performed using one-way analysis of variance (ANOVA) (* *p* < 0.05, ** *p* < 0.01, *** *p* < 0.001). slm = standard liters per minute; ArP = argon plasma (typical setup without adapter).

Up to this point, MoNoS-complemented gas plasma treatment did not improve anticancer efficacy in vitro or in ovo. In the kINPen, the gas plasma discharge in noble feed gas conditions requires 0.1–1% ambient air diffusion in the active plasma zone [16]. However, in ovo kINPen treatment at 5 slm caused CAM rupture and severe bleeding (data not shown), making this treatment scheme not feasible. MoNoS adapters, however, enable such treatment at 5 slm (design point of the MoNoS) without causing major irritation (see below) (Figure 5a). Strikingly, MoNoS I kINPen exposure significantly increased tumor toxicity compared to regular kINPen treatment (ArP) (Figure 5b). This was confirmed by luminescence measurements (Figure 5c) that were found to correlate significantly with the obtained tumor weight (Figure 5d). Surface expression or release of so-called damage-associated molecular patterns (DAMPs) by dying cancer cells can increase their immunogenicity as outlined in the immunogenic cell death (ICD) concept [18]. Tumor therapies aiming at activating the immune system aid in mounting anti-tumor responses and have become increasingly important in clinical oncology. Flow cytometric analysis of tumor single-cell suspensions revealed a significant increase in surface expression of key ICD markers heat shock protein (HSP)70, HSP90, and calreticulin (CRT; Figure 5e) in the ^low^DPR treatment group (Figure 5f). This was paralleled by a notably altered chemokine and cytokine release profile in ^low^DPR conditions compared to the regular kINPen (ArP) exposure (Figure 5g).

### 3.4. MoNoS-Complemented kINPen Treatment Was Well-Tolerated in the HET-CAM Assay

**Figure 6:**
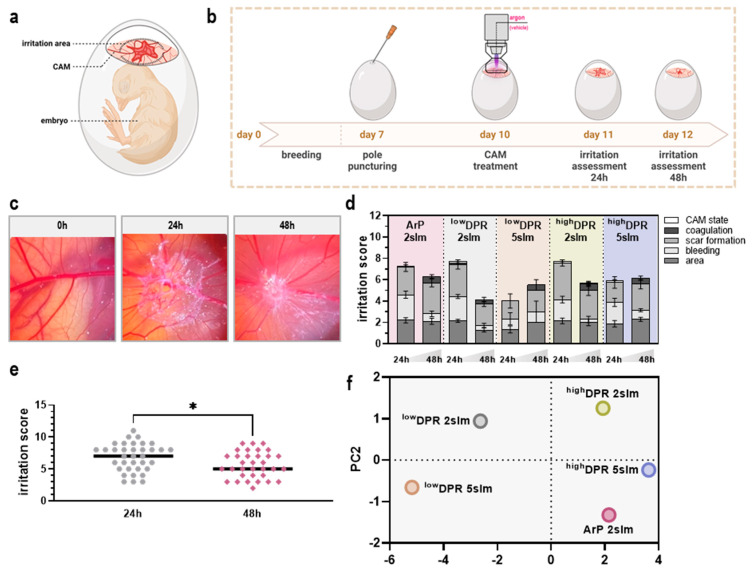
**HET-CAM irritation assay after gas plasma treatment.** (**a**,**b**) Schematic overview of the HET-CAM assay (**a**) and experimental procedure (**b**); (**c**) representative CAM irritations 24 h and 48 h after gas plasma exposure; (**d**) individual scores across all gas plasma groups and two assessment time points; (**e**) cumulative scores across all gas plasma treatment times after 24 h and 48 h; (**f**) principal component analysis of in ovo toxicity and irritation data. ArP = argon plasma (typical setup without adapter); ^low^DPR = low dynamic pressure reduction; ^high^DPR = high dynamic pressure reduction (* *p* < 0.05).

The HET-CAM irritation assay [19] was adapted in this study as a benchmark test to assess the tissue tolerability of regular kINPen argon plasma (ArP) versus MoNoS-complemented kINPen treatment (Figure 6a). After an initial breeding period, eggs were punctured and carefully windowed following gas plasma treatment on ED 10. Tissue tolerability was scored after treatment groups were randomized and blinded (Figure 6b) according to in-house scoring parameters 24 h and 48 h after treatment (Figure 6c). Interestingly, ^low^DPR kINPen treatment gave superior irritation scores concerning the majority of parameters (Figure 6d). In all kINPen conditions, a good remission could be observed after 48 h (Figure 6e). Differences among the applied flow shaping systems were underlined by a notably different principal component 1 (Figure 6f) highlighting that especially effects on the secretion profile mainly contribute to the distinct treatment outcomes (Table A1).

## 4. Discussion

Placed in the heart of applied redox biology, ROS-based therapy approaches such as medical gas plasmas exploit the dual role of ROS in physiological signaling pathways as outlined in the concept of hormesis [19]. The unique feature of this technology constitutes the simultaneous generation of a multitude of highly reactive ROS at body temperature. Initially, gas plasma therapy was approved as a novel physics-based approach for treating chronic wounds [20]. However, in recent years, its successful application in the palliation of head and neck cancer patients further stimulated promising preclinical studies in gas plasma oncology [10]. Still, the obligate need for repetitive treatment cycles limits the application of gas plasma-derived ROS mainly to superficially growing cancers. In addition, intraoperative treatment of surgical tumor margins might serve as a promising strategy to reduce relapse risks caused by remaining micrometastases at the section margins [21].

Despite the diversity of available technical configurations, gas plasma devices most commonly used in biomedical research are either dielectric barrier discharges (DBDs) or gas plasma jets [22,23]. DBDs are more advantageous in the larger treatment area covered than a single jet. In addition, DBD devices often come with stronger fluxes of charged species and electric fields, and many require the target as a third electrode [24]. In the case of gas plasma jets, their advantage delineates their shortcoming at the same time. The small surface contact area enables treatment with high flexibility, which is beneficial for locally restricted, superficially growing cancers or tumor margins in a surgical setting. Supported by gas flow-driven expansion of the gas plasma outside the nozzle, gas plasma jets can penetrate small cavities and structures in a rough and fissured tumor bed [25,26]. However, gas plasma jet treatment times linearly increase with the treatment area needed to be covered. To overcome this limitation and homogenously increase the nominal ROS deposition area while maintaining plasma jets’ flexibility, a Modular Nozzle System was designed in-house, a 3D-printable, modular (ceramic) adapter system for plasma jets, in our case, the kINPen. Due to the self-priming ability of the MoNoS, a stable air–plasma ratio within the active plasma zone for efficient species formation can be achieved. After effluent interaction, a deflector plate is placed into the gas flow. This plate can have a multitude of case-adaptive shapes and properties. In this study, two sieve-like versions were used: one with low and one with high dynamic pressure reduction. However, both adapters partially allow ROS to pass through and/or sideways of the plates.

At modest feed gas fluxes (2 slm), ROS deposition was lower in liquids with both MoNoS adapters, accompanied by limited anticancer efficacy compared to regular kINPen treatment. This is comprehensible for two reasons. First, the gas flow momentum actively drives species into the liquid interphase, inducing liquid convection and providing a continuously renewed liquid volume in regular gas plasma treatments [16]. Second, the volume flow of the feed gas defines the amount of air diffusion into itself. For regular kINPen treatment without MoNoS, both effects increase with increasing gas flow. Using a MoNoS system, this behavior can be designed to the purpose: depending on the deflector plate variant, the stirring effect can be alternated between zero and full due to momentum reduction at any flow rate. The combination of self-priming ability and version (shape) of the ambient intake of MoNoS modulates the air–plasma ratio and hence reactive species production. In the case of pure argon, ROS in the gas phase are generated due to diffusion of ambient air species [27]. Indirectly, an increased gas plasma temperature at lower feed gas fluxes also multiplies the generation of nitric oxide (NO) as a consequence of reduced ozone (O_3_) formation [28]. At the same time, diffusion of ambient species might also lead to collisional quenching, resulting in photo-absorption of VUV radiation generated inside the core gas plasma and, consequently, intensity dips in Ar2* excimers or scavenging of atomic oxygen by ambient air oxygen. However, this is considered irrelevant for most applications [29]. It should be noted that concluding on differing ROS gas plasma chemistries driven by typical kINPen compared to MoNoS-complemented operation is challenging. Disregarding differences concerning gas versus liquid phase ROS, the expected advantage of the MoNoS adapters is the homogenous distribution of ROS in a nominal increased surface area, as well as the ability to treat sensitive surfaces at high flow rates while still having beneficial effects from regular kINPen treatment. Due to liquid convection with regular kINPen treatment, the surface area is artificially enhanced with typical but not MoNoS-complemented kINPen operations, limiting direct comparison between both regimes further.

At 5 slm, ^low^DPR kINPen treatment augmented anti-tumor efficacy above that observed with typical kINPen at similar exposure times. This was paralleled by upregulated expression of pro-immunogenic surface markers and a significantly altered cytokine and chemokine release profile. Engaging the immune system in oncological therapy regimes has been a breakthrough in recent years [30,31]. Extensive research has stimulated the development of diverse strategies to counteract the ability of malignant cells to design immunosuppressive, tumor-promoting microenvironments [32,33]. The notion that some chemotherapeutic agents induce ecto-expression or release of markers that increase the adjuvanticity of cell death has formed the concept of immunogenic cell death (ICD) [18,34]. Similar observations have been made concerning medical gas plasmas [10] and other ROS-based therapy approaches considered bona fide ICD inducers alike [35]. The superior immunogenicity of ^low^DPR-complemented kINPen tumor treatment was further characterized by an altered chemokine and cytokine release profile. In this regard, a significant increase in lymphocyte-activating IL2 was observed along with inflammasome-related IL1β and IL18, which play dual roles in cancer progression and anti-tumor immune responses [36,37,38].

Diffusion distances and reactivity of species generated in the gas phase limit treatment efficacies at larger distances towards a respective target. This must be considered when interpreting MoNoS-complemented kINPen treatment, as the jet is not in direct contact with its target. In part, ROS generated by gas plasmas have very short lifetimes, including ^.^OH with a half-life in biological systems of about 1 ns [39], peroxynitrite (ONOO^−^) with a half-life of about 1 s [40], and superoxide and hydroperoxyl radicals with half-lives of <10 s in aqueous solutions [41]. Unfortunately, directly tracing species trajectories into tissues, such as tumor organoids in our study, is complex, especially with multi-ROS approaches such as gas plasmas. However, the superior reduction of tumor burden in ovo after MoNoS-complemented kINPen treatment at clinically relevant feed gas fluxes (5 slm) underlines the therapeutic efficacy of the treatment.

Novel therapies must be efficient and safe. Previous studies have convincingly demonstrated the safety of gas plasma treatment [20]. Genotoxic events are absent, regardless of feed gas settings [42] and model system (e.g., in ovo) investigated [43]. A one-year follow-up in mice showed a lack of gas plasma-induced preneoplastic or neoplastic lesions in treated wounds [44]. Similar observations have been made in a wound patient cohort lacking malignant transformation, inflammatory reactions, or pathological modifications 5 years after gas plasma therapy [45]. In addition, repeated gas plasma exposure of the oral mucosa over 12 months was not carcinogenic in mice [46]. *In vitro*, anti- but not pro-metastatic effects have been found following kINPen exposure [47]. Notably, MoNoS-complemented kINPen treatment was well-tolerated in the HET-CAM model, outlining its presumably safe application while maintaining high therapeutic efficacy.

## 5. Conclusions

We aimed to analyze the impact of flow shaping on the anticancer efficacy of the kINPen gas plasma treatment. For this, a 3D-printed Modular Nozzle System (MoNoS) was used, reducing feed gas pressure on target surfaces, increasing nominal ROS deposition area, and stabilizing air–plasma ratios, thus ROS production in a self-driven way, for optimized ROS production. At clinically relevant feed gas fluxes, MoNoS-adapted kINPen treatment significantly enhanced anti-tumor effects while maintaining excellent tolerability. These results demonstrate the feasibility of designing and using flow-shaping adapters (such as the MoNoS) for gas plasma jet enhancement in oncology and potentially in other medical fields, such as wound healing.

## Data Availability

The underlying data can be received from the corresponding author upon reasonable request.

## Appendix A

**Table A1:** PC scores of loadings. PC = principal component.

	PC1	PC2
**weight**	0.905	0.035
**luminescence**	0.620	0.739
**irritation score**	0.788	0.189
**IL1β**	−0.989	−0.105
**IP-10**	−0.989	−0.105
**IL2**	−0.989	−0.105
**GM-CSF**	−0.989	−0.120
**G-CSF**	−0.750	0.569
**IL6**	−0.965	−0.226
**HGF**	−0.973	0.228
**IFNγ**	−0.987	0.142
**ENA-78**	−0.959	0.202
**IL18**	−0.985	0.145
**GROα**	−0.996	0.075
**PDFG-aa**	−0.961	0.164
**VEGF**	−0.949	−0.073

## References

[1] Hanahan D., Weinberg R.A. (2011). Hallmarks of cancer: The next generation. Cell.

[2] Kwon J., Lee S.R., Yang K.S., Ahn Y., Kim Y.J., Stadtman E.R., Rhee S.G. (2004). Reversible oxidation and inactivation of the tumor suppressor PTEN in cells stimulated with peptide growth factors. Proc. Natl. Acad. Sci. USA.

[3] Gopalakrishna R., Jaken S. (2000). Protein kinase C signaling and oxidative stress. Free Radic. Biol. Med..

[4] Warburg O. (1956). On the origin of cancer cells. Science.

[5] Alpha-Tocopherol B.C.C.P.S.G. (1994). The effect of vitamin E and beta carotene on the incidence of lung cancer and other cancers in male smokers. N. Engl. J. Med..

[6] Klein E.A., Thompson I.M., Tangen C.M., Crowley J.J., Lucia M.S., Goodman P.J., Minasian L.M., Ford L.G., Parnes H.L., Gaziano J.M. (2011). Vitamin E and the risk of prostate cancer: The Selenium and Vitamin E Cancer Prevention Trial (SELECT). JAMA.

[7] Shen B., He P.J., Shao C.L. (2013). Norcantharidin induced DU145 cell apoptosis through ROS-mediated mitochondrial dysfunction and energy depletion. PLoS ONE.

[8] Brenneisen P., Reichert A.S. (2018). Nanotherapy and Reactive Oxygen Species (ROS) in Cancer: A Novel Perspective. Antioxidants.

[9] Hak A., Ravasaheb Shinde V., Rengan A.K. (2021). A review of advanced nanoformulations in phototherapy for cancer therapeutics. Photodiagnosis Photodyn. Ther..

[10] Bekeschus S., Clemen R. (2022). Plasma, cancer, immunity. J. Phys. D Appl. Phys..

[11] Bruggeman P.J., Bogaerts A., Pouvesle J.M., Robert E., Szili E.J. (2021). Plasma–liquid interactions. J. Appl. Phys..

[12] Metelmann H.R., Seebauer C., Rutkowski R., Schuster M., Bekeschus S., Metelmann P. (2018). Treating cancer with cold physical plasma: On the way to evidence-based medicine. Contrib. Plasma Phys..

[13] Miebach L., Freund E., Clemen R., Weltmann K.D., Metelmann H.R., von Woedtke T., Gerling T., Wende K., Bekeschus S. (2022). Conductivity augments ROS and RNS delivery and tumor toxicity of an argon plasma jet. Free Radic. Biol. Med..

[14] Miebach L., Berner J., Bekeschus S. (2022). In ovo model in cancer research and tumor immunology. Front. Immunol..

[15] Bender C., Partecke L.I., Kindel E., Doring F., Lademann J., Heidecke C.D., Kramer A., Hubner N.O. (2011). The modified HET-CAM as a model for the assessment of the inflammatory response to tissue tolerable plasma. Toxicol. Vitr..

[16] Reuter S., von Woedtke T., Weltmann K.D. (2018). The kINPen-a review on physics and chemistry of the atmospheric pressure plasma jet and its applications. J. Phys. D Appl. Phys..

[17] Freund E., Spadola C., Schmidt A., Privat-Maldonado A., Bogaerts A., von Woedtke T., Weltmann K.D., Heidecke C.D., Partecke L.I., Kading A. (2020). Risk Evaluation of EMT and Inflammation in Metastatic Pancreatic Cancer Cells Following Plasma Treatment. Front. Phys..

[18] Kroemer G., Galluzzi L., Kepp O., Zitvogel L. (2013). Immunogenic cell death in cancer therapy. Annu. Rev. Immunol..

[19] Renschler M.F. (2004). The emerging role of reactive oxygen species in cancer therapy. Eur. J. Cancer.

[20] Bekeschus S., von Woedtke T., Emmert S., Schmidt A. (2021). Medical gas plasma-stimulated wound healing: Evidence and mechanisms. Redox Biol..

[21] Chen G., Chen Z., Wang Z., Obenchain R., Wen D., Li H., Wirz R.E., Gu Z. (2021). Portable air-fed cold atmospheric plasma device for postsurgical cancer treatment. Sci. Adv..

[22] Brandenburg R. (2017). Dielectric barrier discharges: Progress on plasma sources and on the understanding of regimes and single filaments. Plasma Sources Sci. Technol..

[23] Winter J., Brandenburg R., Weltmann K.D. (2015). Atmospheric pressure plasma jets: An overview of devices and new directions. Plasma Sources Sci. Technol..

[24] Kuchenbecker M., Bibinov N., Kaemlimg A., Wandke D., Awakowicz P., Viol W. (2009). Characterization of DBD plasma source for biomedical applications. J. Phys. D Appl. Phys..

[25] Weltmann K.D., Brandenburg R., von Woedtke T., Ehlbeck J., Foest R., Stieber M., Kindel E. (2008). Antimicrobial treatment of heat sensitive products by miniaturized atmospheric pressure plasma jets (APPJs). J. Phys. D Appl. Phys..

[26] Isbary G., Morfill G., Zimmermann J., Shimizu T., Stolz W. (2011). Cold atmospheric plasma: A successful treatment of lesions in Hailey-Hailey disease. Arch. Dermatol..

[27] Lu X., Naidis G.V., Laroussi M., Reuter S., Graves D.B., Ostrikov K. (2016). Reactive species in non-equilibrium atmospheric-pressure plasmas: Generation, transport, and biological effects. Phys. Rep. Rev. Sect. Phys. Lett..

[28] Iseni S., Zhang S., van Gessel A.F.H., Hofmann S., van Ham B.T.J., Reuter S., Weltmann K.D., Bruggeman P.J. (2014). Nitric oxide density distributions in the effluent of an RF argon APPJ: Effect of gas flow rate and substrate. New J. Phys..

[29] Reuter S., Winter J., Schmidt-Bleker A., Schroeder D., Lange H., Knake N., Schulz-von der Gathen V., Weltmann K.D. (2012). Atomic oxygen in a cold argon plasma jet: TALIF spectroscopy in ambient air with modelling and measurements of ambient species diffusion. Plasma Sources Sci. Technol..

[30] Park J.C., Krishnakumar H.N., Saladi S.V. (2022). Current and Future Biomarkers for Immune Checkpoint Inhibitors in Head and Neck Squamous Cell Carcinoma. Curr. Oncol..

[31] Lang F., Schrors B., Lower M., Tureci O., Sahin U. (2022). Identification of neoantigens for individualized therapeutic cancer vaccines. Nat. Rev. Drug Discov..

[32] Tarantino P., Barroso-Sousa R., Garrido-Castro A.C., McAllister S.S., Guerriero J.L., Mittendorf E., Curigliano G., Tolaney S.M. (2022). Understanding resistance to immune checkpoint inhibitors in advanced breast cancer. Expert Rev. Anticancer Ther..

[33] Vitale I., Shema E., Loi S., Galluzzi L. (2021). Intratumoral heterogeneity in cancer progression and response to immunotherapy. Nat. Med..

[34] Obeid M., Panaretakis T., Joza N., Tufi R., Tesniere A., van Endert P., Zitvogel L., Kroemer G. (2007). Calreticulin exposure is required for the immunogenicity of gamma-irradiation and UVC light-induced apoptosis. Cell Death Differ..

[35] Adkins I., Fucikova J., Garg A.D., Agostinis P., Spisek R. (2014). Physical modalities inducing immunogenic tumor cell death for cancer immunotherapy. Oncoimmunology.

[36] Rebe C., Ghiringhelli F. (2020). Interleukin-1beta and Cancer. Cancers.

[37] Hernandez R., Poder J., LaPorte K.M., Malek T.R. (2022). Engineering IL-2 for immunotherapy of autoimmunity and cancer. Nat. Rev. Immunol..

[38] Baker K.J., Houston A., Brint E. (2019). IL-1 Family Members in Cancer; Two Sides to Every Story. Front. Immunol..

[39] Sies H. (1993). Strategies of antioxidant defense. Eur. J. Biochem..

[40] Beckman J.S., Beckman T.W., Chen J., Marshall P.A., Freeman B.A. (1990). Apparent hydroxyl radical production by peroxynitrite: Implications for endothelial injury from nitric oxide and superoxide. Proc. Natl. Acad. Sci. USA.

[41] Marklund S. (1976). Spectrophotometric study of spontaneous disproportionation of superoxide anion radical and sensitive direct assay for superoxide dismutase. J. Biol. Chem..

[42] Bekeschus S., Schmidt A., Kramer A., Metelmann H.R., Adler F., von Woedtke T., Niessner F., Weltmann K.D., Wende K. (2018). High throughput image cytometry micronucleus assay to investigate the presence or absence of mutagenic effects of cold physical plasma. Environ. Mol. Mutagen..

[43] Kluge S., Bekeschus S., Bender C., Benkhai H., Sckell A., Below H., Stope M.B., Kramer A. (2016). Investigating the Mutagenicity of a Cold Argon-Plasma Jet in an HET-MN Model. PLoS ONE.

[44] Schmidt A., Woedtke T.V., Stenzel J., Lindner T., Polei S., Vollmar B., Bekeschus S. (2017). One Year Follow-Up Risk Assessment in SKH-1 Mice and Wounds Treated with an Argon Plasma Jet. Int. J. Mol. Sci..

[45] Rutkowski R., Daeschlein G., von Woedtke T., Smeets R., Gosau M., Metelmann H.R. (2020). Long-term Risk Assessment for Medical Application of Cold Atmospheric Pressure Plasma. Diagnostics.

[46] Evert K., Kocher T., Schindler A., Müller M., Müller K., Pink C., Holtfreter B., Schmidt A., Dombrowski F., Schubert A. (2021). Repeated exposure of the oral mucosa over 12 months with cold plasma is not carcinogenic in mice. Sci. Rep..

[47] Bekeschus S., Freund E., Spadola C., Privat-Maldonado A., Hackbarth C., Bogaerts A., Schmidt A., Wende K., Weltmann K.D., von Woedtke T. (2019). Risk Assessment of kINPen Plasma Treatment of Four Human Pancreatic Cancer Cell Lines with Respect to Metastasis. Cancers.

